# Enhancement of genetic potential for soil carbon and nitrogen cycling by organic fertilizer substitution improves the ecological environment for licorice cultivation

**DOI:** 10.3389/fmicb.2026.1758116

**Published:** 2026-05-12

**Authors:** Guochen Liao, Jiancai Xiao, Boyu Zhang, Sheng Wang, Xiufu Wan, Chengcai Zhang, Chaogeng Lyu, Binbin Yan, Yuping Zhao, Chuanzhi Kang, Yufei Zhang, Feng Yuan, Zhe Zhao, Yunhua Chen, Lanping Guo, Yan Zhang

**Affiliations:** 1 School of Chinese Medicine, Beijing University of Chinese Medicine, Beijing, China; 2 National Key Laboratory for Quality Ensurance and Sustainable Use of Dao-Di Herbs, National Resource Center for Chinese Materia Medica, China Academy of Chinese Medical Sciences, Beijing, China; 3 Laboratory of Molecular Biology and Bovine Breeding, Institute of Animal Science, Chinese Academy of Agricultural Sciences, Beijing, China; 4 Tianjin Research Institute of Construction Machinery Co., Ltd., Tianjin, China; 5 Sinomach Sensing Technology Co., Ltd., Shenyang, Liaoning, China; 6 School of Biomedicine, Beijing City University, Beijing, China; 7 Traditional Therapy Center, Beijing Hongyitang Chinese Medicine Hospital, Beijing, China

**Keywords:** biogeochemical cycle, *Glycyrrhiza uralensis*, metagenomics, microbial functional genes, rational fertilization, rhizosphere vs. bulk soil

## Abstract

**Background:**

Excessive chemical fertilizer application has become a core bottleneck restricting the green and sustainable cultivation of *Glycyrrhiza uralensis* (licorice). Partial organic fertilizer substitution can improve soil microecology and licorice growth traits, yet its regulatory effects on microbial functional genes mediating soil carbon (C) and nitrogen (N) cycling remain unclear.

**Results:**

Using metagenomic sequencing, we investigated the effects of six fertilization regimes [100% organic fertilizer (OF100), 100% chemical fertilizer (OF0), and organic–inorganic combinations (OF25, OF50, OF75)] on the genetic potential of soil C and N cycling, as well as soil properties and licorice growth traits in bulk and rhizosphere soils of licorice. Organic substitution significantly altered the abundance of C and N cycling-related functional genes: OF100 significantly increased the abundance of genes associated with methane oxidation (*pmoA/amoA*), carbon degradation (*pel*, *cbh*) and nitrification (pmoB/amoB), while OF0 significantly upregulated the methanogenesis-related gene mttA and downregulated nitrogen degradation genes; optimized fertilization (OF50) significantly reduced the abundance of genes linked to excessive carbon degradation (*malZ*) and nitrogen loss genetic potential (*nirK*), and markedly increased the abundance of genes for carbon fixation (*pccA*) and nitrogen mineralization (*GDH*). PERMANOVA revealed that soil compartment (bulk vs. rhizosphere) explained 62.87% of the total variation in functional gene profiles, which was 5.67 times higher than the contribution of fertilization regime (11.10%).

**Conclusion:**

Rational organic–inorganic fertilization effectively regulates soil microbial functional genes related to C and N cycling, optimizes soil nutrient cycling potential, reduces nutrient loss risk, and enhances nutrient supply efficiency for licorice growth. These findings provide a scientific basis for fertilizer management optimization and sustainable cultivation of licorice.

## 1 Introduction

In recent decades, agricultural intensification in China has led to excessive fertilizer inputs without proportional increases in crop productivity, resulting in severe soil degradation, reduced nutrient use efficiency, and non-point source pollution, which pose major challenges to the sustainable development of agroecosystems (Jiao et al., 2016; Wang et al., 2020). This issue is particularly prominent in the cultivation of medicinal plants, as the accumulation of bioactive components in medicinal plants is highly dependent on soil microecological conditions, and excessive chemical fertilizer input often leads to quality decline and environmental risks (Egamberdieva et al., 2017; Xiao et al., 2024). *Glycyrrhiza uralensis* (licorice), a widely used traditional Chinese medicinal material with homology of medicine and food, produces core bioactive compounds such as glycyrrhizic acid and isoliquiritin. The synthesis of these secondary metabolites is closely related to soil nutrient supply genetic potential, which is mainly driven by microbially mediated biogeochemical processes (Goudarzi et al., 2024; Xiao et al., 2024).

Organic fertilizer application has been widely adopted as a core green cultivation measure for *Glycyrrhiza uralensis* in recent years, and has become an indispensable technical approach to alleviate the negative impacts of excessive chemical fertilizer use. A growing number of practices have verified that organic amendments (e.g., sheep manure, fermented herbal medicine residues, and functional microbial organic fertilizers) can effectively improve soil physical and chemical properties, enhance soil microbial activity, and promote the root growth and active ingredient accumulation of licorice, which highly aligns with the national strategy of chemical fertilizer reduction and green high-quality development for medicinal plants (Qiu et al., 2025; Abudurezike et al., 2024). The developmental trend of organic fertilization in licorice cultivation has gradually shifted from simple single application of farmyard manure to rational organic–inorganic combined application, from yield-oriented management to the synergistic improvement of yield, medicinal quality and soil health, and from conventional extensive management to precise regulation targeting rhizosphere microecology and nutrient cycling (Egamberdieva et al., 2021). Despite the remarkable progress in organic fertilizer application for licorice, a series of practical and scientific constraints still restrict its large-scale standardized application. In field production, the uneven quality and incomplete maturity of organic raw materials frequently lead to unbalanced soil nutrient supply, and the lack of universal optimal fertilization ratios across different planting regions and soil types makes it difficult to achieve precise nutrient management (Dong et al., 2021). In scientific research, most existing studies only focus on the superficial responses of licorice growth traits and basic soil physicochemical indexes, while the internal mechanism of organic fertilizer regulating soil carbon and nitrogen coupling cycling remains poorly understood (Zhang et al., 2022). In particular, there is still a lack of systematic analysis on the changes of microbial functional gene profiles related to carbon and nitrogen cycling, which is the key scientific issue limiting the clarification of nutrient turnover and the optimization of precision fertilization for licorice. Our previous study confirmed that partial organic substitution improved licorice yield and quality by optimizing soil properties and enhancing microbial diversity (Xiao et al., 2025), but it only explored the taxonomic composition of microbial communities and failed to reveal the regulatory mechanism at the functional gene level. Therefore, this study focuses on the response of soil carbon and nitrogen cycling functional genes to different fertilization regimes, aiming to address the above scientific bottleneck and provide a theoretical basis for efficient and green fertilizer management in licorice cultivation.

Carbon (C) and nitrogen (N) cycling are core coupled biogeochemical processes in terrestrial ecosystems, and their rates and directions are largely regulated by soil microbial communities (Duan et al., 2025; Kuypers et al., 2018). Three microbial-mediated processes are particularly critical for licorice cultivation: (1) methane metabolism, including methanogenesis and methane oxidation, which affects soil carbon sequestration genetic potential and greenhouse gas emissions; (2) denitrification, which converts soil nitrate into gaseous nitrogen (N₂O, N₂), reduces nitrogen use efficiency, and further affects the synthesis of nitrogen-containing secondary metabolites in licorice; (3) nitrogen mineralization and fixation, which control the conversion of organic nitrogen and atmospheric N₂ into plant-available inorganic nitrogen, and can reduce the dependence on chemical nitrogen fertilizers. These processes occur in both bulk soil and rhizosphere microhabitats, and their functional potential is directly shaped by fertilization management and plant–microbe interactions, ultimately affecting licorice productivity, quality, and the ecological sustainability of the cultivation system (Tong et al., 2025).

Previous studies have shown that long-term excessive chemical fertilizer application may increase the genetic potential of soil C and N loss by stimulating the abundance of denitrification and methanogenesis-related genes, while organic fertilizer application can provide diverse carbon substrates for microbial growth, stabilize soil C and N cycling processes, and may improve the functional potential of beneficial nutrient turnover (Lazcano et al., 2021; Yu et al., 2020). In addition, numerous studies have confirmed the strong spatial heterogeneity between bulk and rhizosphere soils: rhizosphere effects can lead to significant differences in microbial community composition and functional gene profiles between the two soil compartments, and the response of rhizosphere and bulk microbial functions to fertilization often shows niche differentiation (Ashish et al., 2024; Sun et al., 2021). However, most existing studies on medicinal plant cultivation focus on the effects of fertilization on soil microbial community composition, and few studies have systematically explored how different organic–inorganic fertilization regimes regulate the functional genes related to key C and N cycling processes (methanogenesis, denitrification, nitrogen fixation, etc.) in bulk and rhizosphere soils of licorice. Meanwhile, the regulatory mechanism of rational fertilization on the coupling of soil C and N cycling at the functional gene level remains to be elucidated. Therefore, this study aims to: (1) evaluate the effects of chemical fertilizer, organic fertilizer, and their combined application on the abundance of microbial functional genes related to C and N cycling (focusing on methane metabolism, denitrification, nitrogen mineralization and fixation) in bulk and rhizosphere soils of licorice; (2) reveal the relative influence of soil compartment (bulk vs. rhizosphere) and fertilization regime on soil microbial functional gene profiles; (3) explore the potential mechanism by which rational fertilization optimizes the genetic potential of soil C and N coupling cycling to support the ecological sustainability of licorice cultivation.

## 2 Materials and methods

### 2.1 Study site

The field experiment was conducted from April 2022 to November 2023 at an agricultural farm in Guyuyuan, Yukou Town, Pinggu District, Beijing, China (117°12′E, 40°07′N). This region is located in the northeastern plain of Beijing, with a warm temperate semi-humid continental monsoon climate. The annual average temperature is 11.7 °C, annual average precipitation is 572.1 mm (concentrated mainly from July to September), annual total sunshine duration is approximately 2579.1 h, and the frost-free period is about 190 days. The terrain is dominated by alluvial–pluvial plains, with high terrain in the northeast and low terrain in the southwest. The soil at the experimental site is loessial soil, classified as cinnamon soil (calcareous brown subtype) in the Chinese genetic soil classification system, and corresponds to calcic Cambisols according to the FAO World Reference Base for Soil Resources (WRB) classification system. The basic physicochemical properties of the topsoil (0–20 cm) before the experiment are presented in Table 1. The area is a typical agricultural region for medicinal plant cultivation, with good drainage. A location map of the study area is provided in Supplementary Figure S1.

**Table 1:** Basic characteristics of the topsoil layer (0–20 cm).

Soil basic properties	
pH	8.5
Total nitrogen (TN, g· kg^−1^)	0.98
Total phosphorus (TP, g· kg^−1^)	0.46
Total potassium (TK, g· kg^−1^)	29.12
Total carbon (TC, g· kg^−1^)	27.61
Soil organic matter (SOM, g· kg^−1^)	14.5

### 2.2 Experimental design

The licorice seedlings used in this study were one-year-old *Glycyrrhiza uralensis* Fisch, provided by Yihong Breeding Cooperative in Hangjinqi, Inner Mongolia. Uniform seedlings with stem length of about 6 cm and main root length of about 20 cm were selected for transplanting.

The field experiment adopted a completely randomized block design with three replications. The entire experimental field was divided into 3 independent blocks, and each block was further divided into 6 equal-sized plots, corresponding to 6 fertilization treatments (OF100, OF75, OF50, OF25, OF0, and CK). Each plot had an area of 4 m^2^ (2 m × 2 m), with a 0.5 m wide isolation belt between adjacent plots to prevent nutrient cross-contamination, and a 50 cm wide buffer zone around the entire experimental area. Within each block, 6 plots were randomly assigned to 6 treatments by lottery method, ensuring that each treatment appeared exactly once in each block to eliminate the interference of soil spatial heterogeneity on the experimental results. The fertilization gradient was set based on the basic soil nutrient content, field management conditions, and traditional fertilization rate for licorice cultivation. The total input of N, P₂O₅ and K₂O was consistent across all fertilization treatments through mole equivalent conversion, and the specific settings of each treatment were as follows: (1) OF100: 100% organic fertilizer (sheep manure) with an application rate of 15,464 kg hm^−2^; (2) OF75: 75% organic fertilizer (11,598 kg hm^−2^) + 25% chemical fertilizer (N 56.25 kg hm^−2^, P_2_O_5_ 56.25 kg hm^−2^, K_2_O 56.25 kg hm^−2^); (3) OF50: 50% organic fertilizer (7,732 kg hm^−2^) + 50% chemical fertilizer (N 112.5 kg hm^−2^, P_2_O_5_ 112.5 kg hm^−2^, K_2_O 112.5 kg hm^−2^); (4) OF25: 25% organic fertilizer (3,866 kg hm^−2^) + 75% chemical fertilizer (N 168.75 kg hm^−2^, P_2_O_5_ 168.75 kg hm^−2^, K_2_O 168.75 kg hm^−2^); (5) OF0: 100% chemical fertilizer (N 225 kg hm^−2^, P_2_O_5_ 225 kg hm^−2^, K_2_O 225 kg hm^−2^); (6) Blank control (CK): no fertilizer was applied, and only conventional field management (such as weeding and irrigation) was carried out. The chemical fertilizers used in the experiment were commercially available products (urea containing 46% N, calcium superphosphate containing 16%P_2_O_5_, and potassium oxide containing 60% K_2_O, purchased from China Chemical Fertilizer Co., Ltd.). The organic fertilizer was sheep manure, and its basic physical and chemical properties were as follows: organic matter 458 g kg^−1^, total nitrogen 15.80 g kg^−1^, total phosphorus 17.78 g kg^−1^, total potassium 14.55 g kg^−1^, alkali-hydrolyzable nitrogen 2.74 g kg^−1^, available phosphorus 1.45 g kg^−1^, available potassium 1.55 g kg^−1^, and pH 7.03. Each plot was planted with 50 licorice seedlings, with a row spacing of 40 cm and a plant spacing of 10 cm. Two weeks before transplanting, the experimental field was deeply plowed and leveled, and surface debris was removed. All fertilizers were applied as base fertilizer at one time: fertilizers were accurately weighed according to the plot area, evenly spread on the soil surface, fully mixed with the 0–20 cm topsoil, and then covered with shallow soil. During the whole growth period, the field management followed the standard agronomic practices for licorice cultivation, with consistent weeding, irrigation and pest control measures across all plots.

### 2.3 Soil sampling

At the licorice harvest stage in November 2023, rhizosphere and bulk soil samples were collected from each experimental plot. An S-shaped sampling method was used, with 15 licorice plants excavated per plot. Loosely attached soil was gently shaken off, and soil tightly adhering to the roots was brushed off to obtain rhizosphere soil, which was then passed through a 2 mm sieve to remove debris. Bulk soil was collected from the 10–20 cm layer surrounding the roots. All sampling tools, including stainless steel shovels, were cleaned with sterile cotton balls between plots to minimize cross-contamination. The 15 individual samples from each plot were thoroughly mixed to form a composite soil sample. Each composite was divided into two portions. One portion was immediately stored on dry ice in the field, then transferred to the laboratory, flash-frozen in liquid nitrogen for 5 min, and stored at −80 °C for metagenomic sequencing. The effects of different fertilization treatments on soil physicochemical properties and licorice agronomic traits and yield are shown in Tables 2, 3, respectively.

**Table 2:** Soil physical and chemical properties of different fertilizer treatments ( $\bar{x}\pm s$ , *n* = 3).

Group	pH	AK/mg kg^−1^	AN/mg kg^−1^	TK/g kg^−1^	TN/g kg^−1^	TP/g kg^−1^	TC/g kg^−1^	SOM/g kg^−1^	SOC/g kg^−1^	EC/ms m^−1^
CK	8.51 ± 0.02a	132 ± 25.5c	69 ± 7.4b	19.97 ± 0.27a	1.01 ± 0.04a	0.48 ± 0.38b	33.1 ± 3.74b	16.4 ± 1.68b	9.52 ± 0.97b	13.0 ± 1.97ab
OF100	8.40 ± 0.12ab	231 ± 35.9a	83 ± 4.6a	20.01 ± 0.18a	1.27 ± 0.19a	0.59 ± 0.28a	46.2 ± 5.57a	19.5 ± 0.95a	11.37 ± 0.54a	14.73 ± 1.33ab
OF75	8.37 ± 014ab	200 ± 19.8ab	75 ± 6.1ab	20.26 ± 0.17a	1.32 ± 0.09a	0.55 ± 0.19ab	44.7 ± 5.28ab	18.7 ± 0.66ab	10.83 ± 0.40ab	14.4 ± 0.26ab
OF50	8.26 ± 0.10bc	163 ± 28.4bc	70 ± 3.7ab	19.81 ± 0.85a	1.24 ± 0.15a	0.51 ± 0.47ab	37.8 ± 3.13ab	17.9 ± 0.88ab	10.39 ± 0.52ab	12.4 ± 0.65a
OF25	8.12 ± 0.06 cd	157 ± 13.8bc	75 ± 2.8ab	19.87 ± 0.33a	1.25 ± 0.07a	0.55 ± 0.25ab	38.7 ± 1.55ab	17.8 ± 0.59ab	10.3 ± 0.36ab	13.8 ± 1.56ab
OF0	7.94 ± 0.09d	128 ± 15.9c	72 ± 5.3ab	19.65 ± 0.27a	1.28 ± 0.16a	0.56 ± 0.68ab	35.4 ± 3.48ab	17.6 ± 1.65ab	10.23 ± 0.96ab	14.9 ± 0.54b

Different lowercase letters within the same column indicate significant differences among treatments at *p* < 0.05 by Duncan’s multiple range test.

**Table 3:** Effects of different fertilizer treatments on character and yield of *G. uralensis* ( $\bar{x}\pm s$ , *n* = 15).

Group	Root length/cm	Root weight/g	D1/cm	D20/cm	Yield/g m^2^
CK	41.23 ± 1.08a	8.13 ± 0.56d	6.79 ± 0.38c	5.74 ± 0.78a	101.47 ± 7.01d
OF100	42.56 ± 1.03a	8.73 ± 0.49 cd	6.96 ± 0.38bc	5.11 ± 0.44a	109.11 ± 6.16 cd
OF75	42.75 ± 2.25a	10.87 ± 0.82ab	8.50 ± 0.11a	5.72 ± 0.21a	135.84 ± 10.27ab
OF50	41.15 ± 1.46a	10.60 ± 0.47abc	7.90 ± 0.41ab	5.93 ± 0.65a	132.50 ± 5.93abc
OF25	41.88 ± 2.93a	12.28 ± 0.38a	8.91 ± 0.84a	5.97 ± 0.33a	153.53 ± 4.69a
OF0	40.03 ± 1.17a	9.14 ± 1.42bcd	7.34 ± 0.14bc	5.11 ± 0.35a	114.24 ± 17.81bcd

Different lowercase letters within the same column indicate significant differences among treatments at *p* < 0.05 by Duncan’s multiple range test.

### 2.4 DNA extraction, metagenomics sequencing, and bioinformatic analysis

Total soil microbial DNA was extracted from 0.5 g fresh soil samples using the FastDNA^®^ SPIN Kit for Soil (MP Biomedicals, United States) according to the manufacturer’s instructions. The concentration and purity of the extracted DNA were detected by a NanoDrop 2000 spectrophotometer (Thermo Scientific, United States), and the integrity of DNA was verified by 1% agarose gel electrophoresis. Qualified DNA samples were used for metagenomic sequencing, with 3 biological replicates for each treatment.

Metagenomic sequencing was performed on the Illumina HiSeq 4000 platform (Illumina Inc., San Diego, CA, United States) by LingEn Biotechnology Co., Ltd. (Shanghai, China), and paired-end sequencing with a read length of 150 bp was adopted. A total of 36 metagenomic datasets (6 treatments × 2 soil compartments × 3 replicates) were obtained in this study. Raw reads were quality-filtered using Fastp (v0.20.0) (Chen, 2023): adapter sequences, reads with length <50 bp, average quality score <20, and sequences containing ambiguous bases (N) were removed to obtain high-quality clean reads. Clean reads were assembled into contigs using MEGAHIT (v1.1.2) with optimized k-mer parameters, and contigs longer than 300 bp were retained for subsequent analysis. Open reading frames (ORFs) were predicted from the retained contigs using MetaGene, and translated into amino acid sequences. All predicted ORFs were clustered into a non-redundant gene catalog using CD-HIT (v4.6.1) with a sequence identity threshold of 95% and a coverage threshold of 90% (Bahram et al., 2018). Clean reads of each sample were aligned to the non-redundant gene catalog using SOAPaligner (v2.2.1), and the abundance of each gene in each sample was calculated based on the number of mapped reads. The gene abundance was normalized by the housekeeping gene *rplB*, which was selected as the internal reference due to its stable transcription level in diverse bacterial taxa and environmental conditions (Wang et al., 2015).

Functional annotation of the non-redundant gene catalog was performed using DIAMOND (v0.8.35) against the Kyoto Encyclopedia of Genes and Genomes (KEGG) database (Buchfink et al., 2015). Genes involved in carbon cycling (carbon fixation, carbon degradation, methane metabolism) and nitrogen cycling (nitrogen fixation, nitrification, denitrification, assimilatory nitrate reduction (ANRA), dissimilatory nitrate reduction to ammonium (DNRA), nitrogen mineralization/degradation) were screened according to the KEGG functional annotation results, and the detailed list of target genes is shown in Supplementary Table S1. For key functional genes with high sequence homology (pmoA/amoA, pmoB/amoB), we performed strict annotation quality control to avoid misclassification: We retained only the annotation results with *e*-value <1 × 10^−50^ and identity >80% for pmoA/amoA and pmoB/amoB genes, to improve the accuracy of annotation.

### 2.5 Statistical analysis

Shapiro–Wilk test and Levene test were used to test the normality and homogeneity of variance of the data, respectively, and non-normally distributed data were log-transformed before statistical analysis. One-way analysis of variance (ANOVA) was used to test the significant differences in soil physicochemical properties, licorice agronomic traits, and functional gene abundance among different fertilization treatments, and Duncan’s multiple range test was used for post-hoc comparison (*p* < 0.05). Wilcoxon rank-sum test was used to analyze the significant differences in functional gene abundance between fertilized treatments and the CK control, and between bulk and rhizosphere soils. Principal coordinates analysis (PCoA) based on Bray–Curtis distance was used to visualize the overall differences in microbial functional gene profiles among different treatments and soil compartments. Analysis of similarities (ANOSIM) was used to test the significance of differences in functional gene profiles between groups. Permutational multivariate analysis of variance (PERMANOVA) was performed using the “vegan” package in R 4.3.0, to quantify the relative contribution of soil compartment and fertilization regime to the variation of microbial functional gene profiles, with 999 permutations.

Spearman correlation analysis was used to explore the relationships between soil physicochemical properties and the abundance of C/N cycling functional genes, as well as the dominant microbial phyla. Correlation heatmaps were drawn using the “pheatmap” package in R 4.3.0. The co-occurrence network of C and N cycling functional genes was constructed using Cytoscape 3.8.2, based on Spearman correlation coefficients (|*r*| > 0.6, *p* < 0.05). All figures were prepared using Origin 2021 and Adobe Illustrator CC 2021.

## 3 Results

### 3.1 Effects of fertilization on soil physicochemical properties and licorice agronomic traits

The effects of different fertilization treatments on soil physicochemical properties are shown in Table 2. Compared with the CK control, fertilization treatments significantly increased soil total nitrogen (TN), total carbon (TC), soil organic matter (SOM) and soil organic carbon (SOC) content (*p* < 0.05). With the increase of organic fertilizer substitution ratio, soil pH showed a gradual upward trend, and the pH of OF100 treatment was significantly higher than that of OF0 treatment (*p* < 0.05). Soil available potassium (AK) content was the highest in OF100 treatment, which was significantly higher than that in OF0 and CK treatments (*p* < 0.05). There was no significant difference in total potassium (TK) content among all treatments (*p* > 0.05). The effects of different fertilization treatments on licorice agronomic traits and yield are shown in Table 3. Compared with CK, all fertilization treatments increased licorice root weight and yield to varying degrees. The yield of OF25 treatment was the highest (153.53 g m^−2^), which was 51.3% higher than that of CK (*p* < 0.05), followed by OF75 and OF50 treatments, while there was no significant difference between OF0 and CK treatments (*p* > 0.05). The root diameter (D1) of OF25, OF75 and OF50 treatments was significantly larger than that of CK and OF0 treatments (*p* < 0.05).

### 3.2 Overall characteristics of microbial functional gene profiles

**Figure 1:**
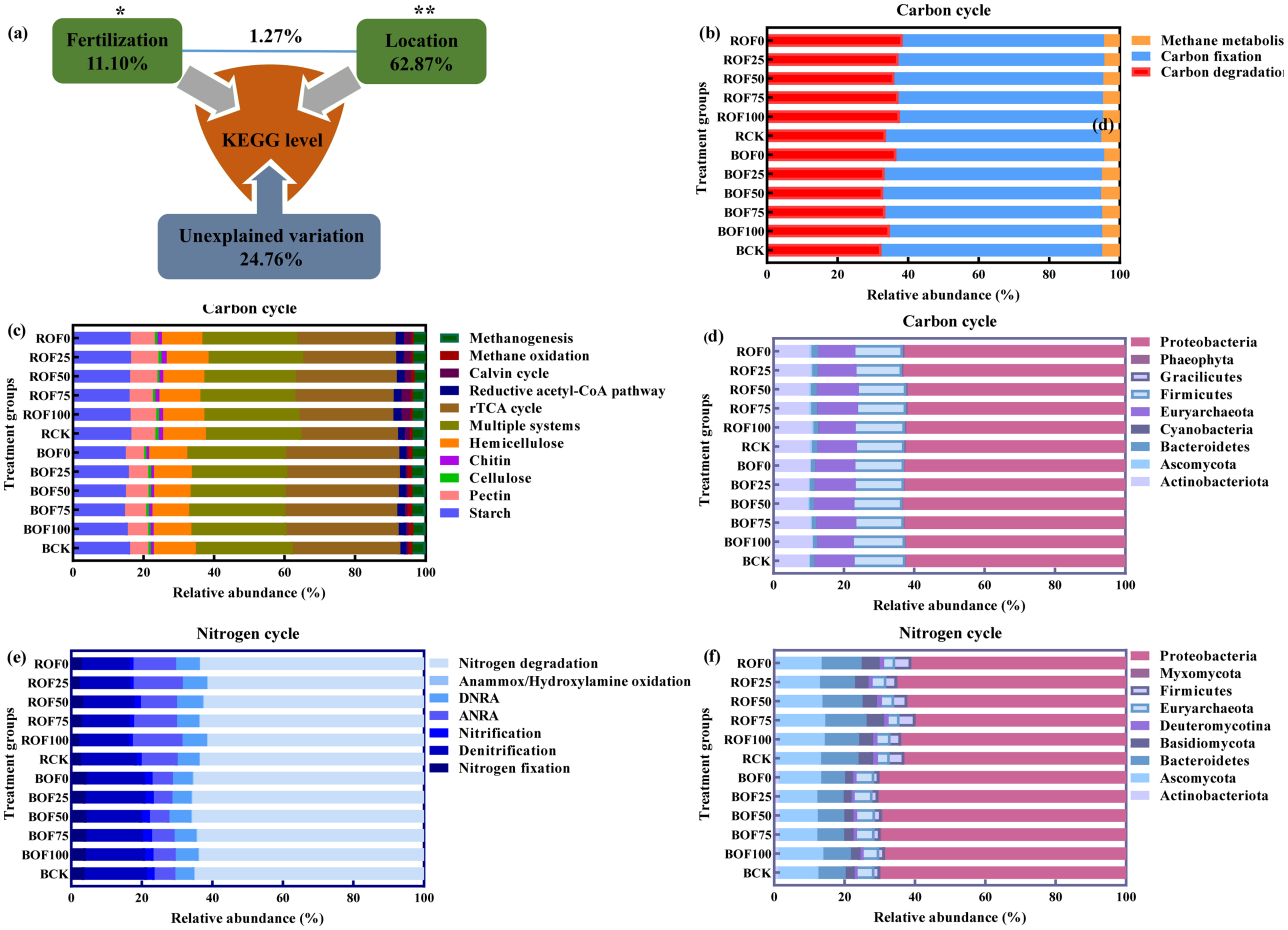
**(a)** PERMANOVA analysis quantifies the effects of fertilization regime and soil compartment. Asterisks indicate significant differences (*p < 0.05 and **p < 0.01). Relative abundance of functional genes involved in carbon **(b,c)** and nitrogen (e) cycling at the functional level, and the corresponding microbial taxa at the phylum level for carbon **(d)** and nitrogen **(f)** cycles.

After quality control and annotation, a total of 24,018 KEGG Orthology (KO) identifiers were obtained from all 36 metagenomic samples. PERMANOVA analysis showed that soil compartment (bulk vs. rhizosphere) was the primary factor driving the variation of overall microbial functional gene profiles, explaining 62.87% of the total variance (*p* = 0.001), while fertilization regime explained 11.10% of the variance (*p* = 0.033) (Figure 1a; Supplementary Table S2).

**Figure 2:**
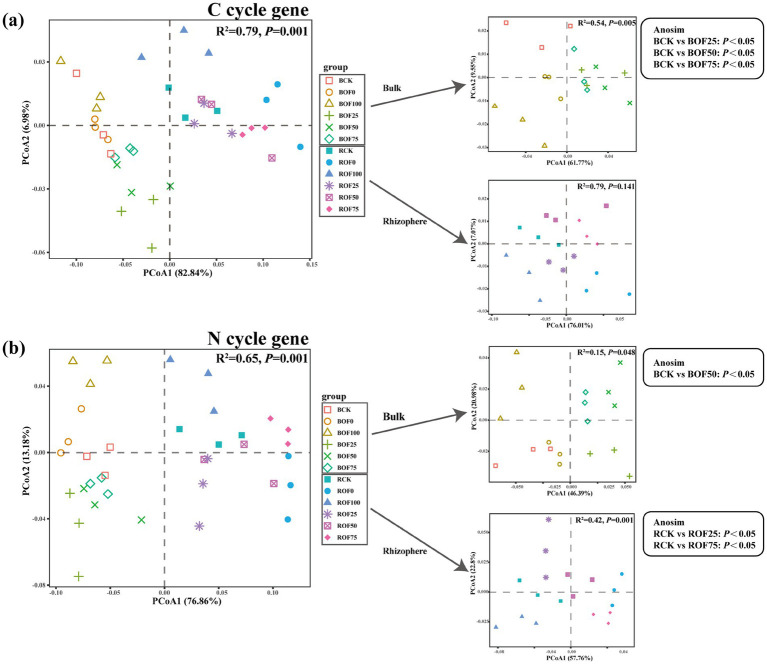
PCoA of soil microbial functional profiles involved in **(a)** carbon and **(b)** nitrogen cycling based on Bray–Curtis distances. Plots compare functional gene composition between fertilization treatments and soil compartments (bulk vs. rhizosphere soil). Inset boxes show ANOSIM statistics for the effects of fertilization and soil compartment.

PCoA based on Bray–Curtis distance showed that the functional gene profiles of C and N cycling were clearly separated between bulk and rhizosphere soils along the first principal axis (PC1), which explained 82.84 and 76.86% of the variance for C and N cycling genes, respectively (Figure 2). ANOSIM analysis further confirmed that there were extremely significant differences in C and N cycling functional gene profiles between bulk and rhizosphere soils (*p* = 0.001). Organic fertilizer substitution treatments significantly affected the C cycling functional gene profile in bulk soil and the N cycling functional gene profile in rhizosphere soil (*p* < 0.05), while there was no significant difference between single fertilizer treatments (OF0, OF100) and CK in the overall functional profile (*p* > 0.05).

### 3.3 Response of carbon cycling functional genes to fertilization regimes

The carbon cycling functional genes were divided into three categories: carbon fixation, carbon degradation, and methane metabolism (Supplementary Table S3). Among them, carbon fixation-related genes had the highest relative abundance, followed by carbon degradation-related genes (Figure 1b). In carbon fixation pathways, genes related to the multi-enzyme system and reductive tricarboxylic acid (rTCA) cycle were more abundant than those related to the reductive acetyl-CoA pathway and Calvin cycle. In carbon degradation pathways, genes related to starch, hemicellulose and pectin degradation had the highest relative abundance across all treatments (Figure 1c).

**Figure 3:**
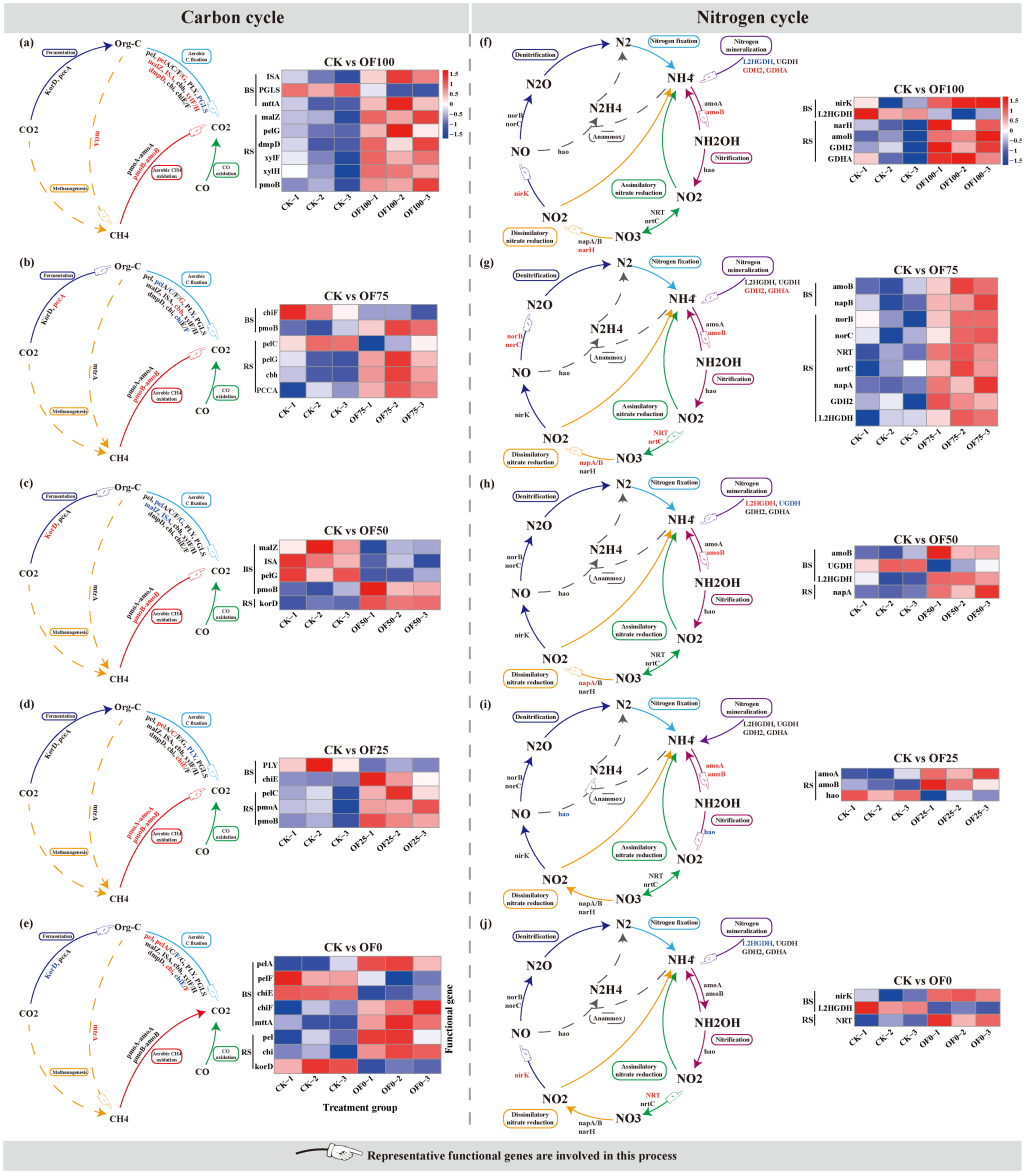
Carbon **(a–e)** and nitrogen **(f–j)** cycling pathways inferred from metagenomic data, comparing fertilized treatments relative to the control (CK). Red/blue genes indicate significant increases/decreases under fertilization. Heatmaps show the relative abundance of differential genes (red = high, blue = low) to complement pathway analysis.

Wilcoxon rank-sum test showed that different fertilization treatments induced significant changes in the abundance of carbon cycling-related functional genes in both bulk and rhizosphere soils, compared with the CK control (Figures 3a–e). All fertilization treatments significantly altered the abundance of carbon degradation-related genes: OF50 treatment significantly down-regulated the abundance of starch degradation gene (*malZ*) and pectin degradation gene (*pel*) (*p* < 0.05), while OF0 treatment significantly up-regulated the abundance of multiple carbon degradation genes (*malZ*, *pelA*, *chiE*) (*p* < 0.05). For methane metabolism, all organic–inorganic combined treatments (OF25-OF75) significantly up-regulated the abundance of pmoA/amoA, pmoB/amoB (methane oxidation/nitrification homologous genes, annotated as methane oxidation-related under strict threshold) (*p* < 0.05), while OF0 treatment had no significant effect on these genes. Notably, the methanogenesis-related gene mttA was significantly up-regulated only in the OF0 treatment, and this change occurred exclusively in bulk soil (*p* < 0.05). The abundance of carbon fixation genes *pccA* and *korD* was significantly up-regulated in the rhizosphere soil under OF75 and OF50 treatments (*p* < 0.05), but significantly down-regulated under OF0 treatment (*p* < 0.05).

Taxonomic annotation showed that the carbon cycling functional genes were mainly derived from Proteobacteria, followed by Firmicutes, Actinobacteriota, Bacteroidota and Acidobacteriota (Figure 1d). Proteobacteria contributed 42.3–58.7% of the total carbon cycling functional genes across all treatments, and the relative contribution of Proteobacteria to carbon fixation and methane oxidation genes was significantly higher in organic fertilizer substitution treatments than in OF0 treatment (*p* < 0.05). Actinobacteriota contributed 11.2–18.6% of the carbon degradation genes, and its contribution was significantly positively correlated with the organic fertilizer substitution ratio (*r* = 0.72, *p* < 0.01).

### 3.4 Response of nitrogen cycling functional genes to fertilization regimes

The nitrogen cycling functional genes were divided into seven processes: nitrogen fixation, denitrification, nitrification, ANRA, DNRA, anaerobic ammonia oxidation/hydroxylamine oxidation, and nitrogen mineralization/degradation (Supplementary Table S3). Among them, nitrogen mineralization/degradation-related genes had the highest relative abundance, followed by denitrification, ANRA, DNRA and nitrogen fixation-related genes (Figure 1e).

Wilcoxon rank-sum test showed that fertilization treatments significantly altered the abundance of nitrogen cycling-related genes in both bulk and rhizosphere soils, and the response patterns of pure chemical fertilizer and organic fertilizer treatments were significantly different (Figures 3f–j). OF0 treatment significantly up-regulated the abundance of denitrification-related gene nirK and ANRA-related gene *NRT* (*p* < 0.05), but significantly down-regulated the abundance of nitrogen mineralization-related gene *GDH* (a gene related to microbial intracellular nitrogen metabolism, associated with nitrogen mineralization potential) (*p* < 0.05). Organic fertilizer treatments (OF75, OF100) significantly up-regulated the abundance of pmoA/amoA、pmoB/amoB (methane oxidation/nitrification homologous genes, annotated as nitrification-related under strict threshold) (*p* < 0.05). OF75 treatment significantly increased the abundance of genes in all nitrogen cycling processes except anaerobic ammonia oxidation (*p* < 0.05), while anaerobic ammonia oxidation-related genes were significantly down-regulated in OF25 treatment (*p* < 0.05).

The distribution of nitrogen cycling functional genes showed significant niche differentiation between bulk and rhizosphere soils: the abundance of nitrogen mineralization-related genes was higher in the rhizosphere soil under high organic substitution ratio treatments (OF50–OF100), while it was higher in bulk soil under low organic substitution ratio treatments (OF0–OF25); nitrification-related genes showed the opposite distribution pattern. Taxonomic annotation showed that the nitrogen cycling functional genes were mainly derived from Proteobacteria, followed by Ascomycota, Bacteroidota, Actinobacteriota and Firmicutes (Figure 2f). Proteobacteria contributed 51.4–65.8% of the total nitrogen cycling functional genes, and its contribution to denitrification and nitrification genes was significantly higher in OF0 treatment than in organic fertilizer treatments (*p* < 0.05). Ascomycota contributed 8.7–15.3% of the nitrogen mineralization-related genes, and its contribution was significantly positively correlated with the organic fertilizer substitution ratio (*r* = 0.68, *p* < 0.01).

### 3.5 Linkages between soil physicochemical properties, microbial taxa and functional genes

**Figure 4:**
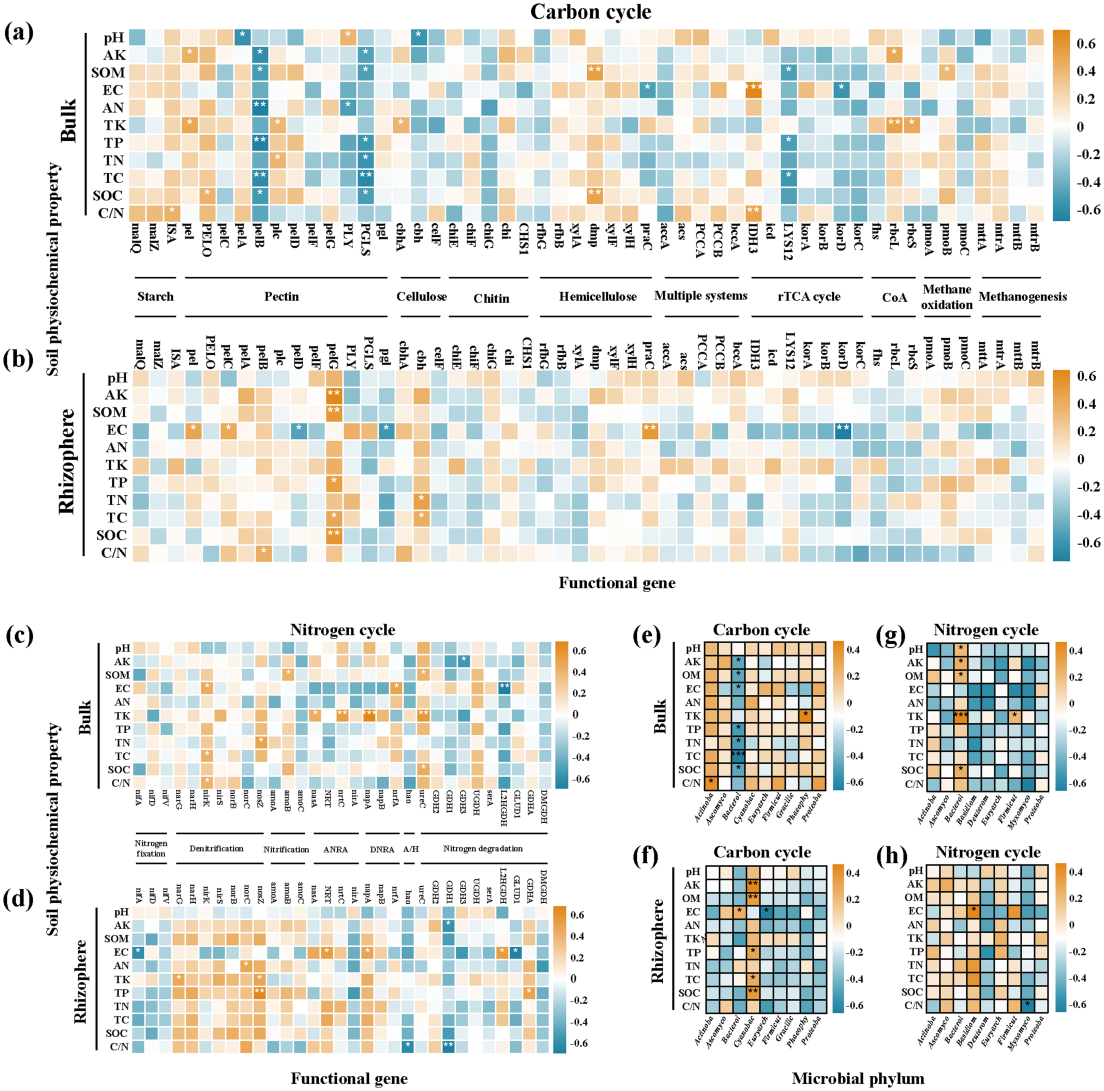
Spearman correlation heatmaps showing relationships between soil physicochemical properties and **(a–d)** C/N cycling functional genes, or **(e–h)** dominant microbial phyla, across different soil microhabitats. Color intensity indicates correlation strength (yellow = positive, blue = negative) (^*^p ≤ 0.05 and ^**^p ≤ 0.01).

Spearman correlation analysis showed that soil physicochemical properties were significantly correlated with the abundance of C and N cycling functional genes, and the correlation patterns were different between bulk and rhizosphere soils (Figures 4a–d). In bulk soil, total potassium (TK) was significantly positively correlated with the abundance of Calvin cycle-related carbon fixation genes *rbcL* and *rbcS* (*p* < 0.05); total phosphorus (TP), AK, TN, SOM were significantly negatively correlated with the abundance of pectin degradation genes *pelB* and *PGLS* (*p* < 0.05). In rhizosphere soil, TP was significantly positively correlated with the abundance of denitrification gene nosZ and nitrogen mineralization gene *GDHA* (*p* < 0.05), while C/N ratio and electrical conductivity (EC) were significantly negatively correlated with the abundance of nitrogen mineralization-related genes (*p* < 0.01). The nirK gene was significantly positively correlated with soil C/N ratio and TC in bulk soil (*p* < 0.05), and TK was significantly positively correlated with ANRA-related gene nrtC and DNRA-related gene napA in bulk soil (*p* < 0.05).

**Figure 5:**
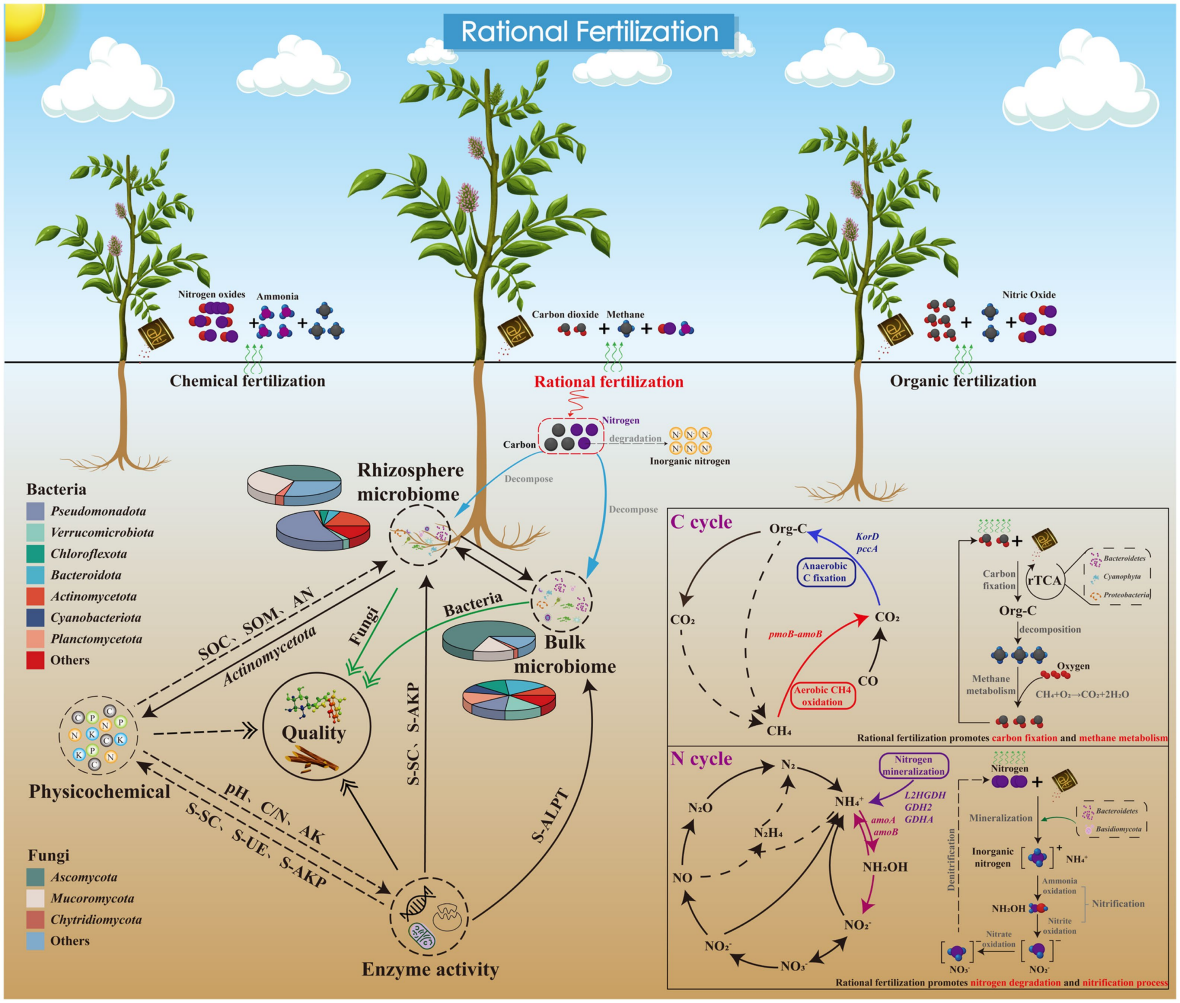
Conceptual model illustrating how optimized fertilization regulates plant–soil–microbiome interactions and C–N cycling. Rational fertilization shapes rhizosphere and bulk soil microbial communities (e.g., Pseudomonadota, Verrucomicrobiota, Ascomycota, Mucoromycota). These microbiomes interact with soil physicochemical properties (pH, C/N, AK, etc.) and extracellular enzyme activities, driving the genetic potential of key C cycling processes (e.g., organic–inorganic C transformation, methane metabolism via KorD and pccA-related pathways) and N cycling pathways (e.g., nitrification, denitrification, nitrogen mineralization regulated by nirK, GDH1/2). Compared to single chemical or organic fertilization, rational fertilization optimizes microbial-mediated C–N coupling potential, and may enhance the genetic potential of soil–plant system functional regulation by coordinating biogeochemical cycling potential and plant–microbe–soil interactions.

For the dominant microbial phyla, Actinobacteriota in bulk soil was significantly negatively correlated with AK, SOM, TC, TP and SOC (*p* < 0.05), while Cyanobacteria in rhizosphere soil was significantly positively correlated with these soil properties (*p* < 0.05) (Figures 4e–h). Actinobacteriota in bulk soil was significantly positively correlated with TK (*p* < 0.01), while the correlation between rhizosphere microbial phyla and soil properties was generally weaker than that in bulk soil (see Figure 5).

## 4 Discussion

### 4.1 Effects of organic fertilizer substitution on the genetic potential of soil C and N cycling

Soil C and N cycling are core biogeochemical processes that maintain soil fertility and support crop growth, and their direction and intensity are largely determined by the functional composition of soil microbial communities (Shah et al., 2019). Metagenomic technology can reveal the genetic potential of microbial communities to participate in nutrient cycling, but it cannot directly reflect the actual rate of biogeochemical processes, which is also affected by gene expression, enzyme activity, and environmental conditions (Nannipieri et al., 2020). In this study, we found that organic fertilizer substitution significantly modulated the abundance of microbial functional genes related to soil C and N cycling in licorice cultivation system, indicating that rational fertilization can alter the genetic potential of microbial-mediated nutrient cycling processes.

For carbon cycling, we found that organic fertilizer substitution treatments increased the abundance of genes related to pectin and cellulose degradation (*pel*, *cbh*), which is consistent with previous studies showing that organic fertilizer input can provide diverse complex carbon substrates, and select for microbial populations with strong lignocellulose degradation capacity (Zhang Q. et al., 2017; Zhang L. et al., 2017). The up-regulation of these carbon degradation genes indicates a higher genetic potential for complex organic matter decomposition, which may enhance the genetic potential of soil organic matter conversion to plant-available nutrients, and provide continuous carbon skeletons and energy for the synthesis of secondary metabolites in licorice (Xiao et al., 2025). Meanwhile, OF50 and OF75 treatments significantly up-regulated the abundance of carbon fixation genes *pccA* and *korD* in the rhizosphere soil, suggesting that rational fertilization may enhance the genetic potential of microbial carbon sequestration genetic potential in the rhizosphere, which is conducive to improving soil structure and long-term soil fertility (Maltas et al., 2018). Notably, OF0 treatment significantly up-regulated the abundance of methanogenesis-related gene *mttA*. This result is consistent with previous findings that long-term chemical nitrogen fertilizer application can increase the abundance of methanogenesis-related genes and the potential of methane emissions, while organic fertilizer substitution may promote methane oxidation and reduce the net greenhouse gas emission potential (Yu et al., 2020). It should be noted that this study only characterized the abundance of related functional genes, and the actual methane emission flux needs to be verified by in-situ gas monitoring in future studies.

For nitrogen cycling, we found that OF0 treatment significantly up-regulated the abundance of denitrification gene nirK and down-regulated the abundance of *GDH* (a gene associated with nitrogen mineralization potential, involved in microbial intracellular nitrogen metabolism). Nitrification is the core process of soil nitrogen conversion, and the up-regulation of *amoA*/*amoB* genes indicates a higher genetic potential for ammonia oxidation, which may improve the genetic potential of soil nitrate nitrogen supply efficiency (Chuckran et al., 2021; Kuypers et al., 2018). The down-regulation of *nirK* gene in OF50 treatment suggests that rational fertilization may reduce the genetic potential of denitrification, thus reducing the risk of gaseous nitrogen loss genetic potential (Kelly et al., 2021). However, we also found that OF100 treatment increased the abundance of some denitrification-related genes, which may be due to the high organic carbon input in pure organic fertilizer treatment, which provides energy for denitrifying microorganisms (Lazcano et al., 2021). This contradictory result indicates that the effect of organic fertilizer on soil nitrogen loss genetic potential is not linear, and moderate organic–inorganic combination is more conducive to balancing nitrogen supply and reducing loss risk.

### 4.2 Linkages between functional genes, microbial taxa and licorice performance

In this study, we found that the C and N cycling functional genes in licorice cultivation soil were mainly derived from Proteobacteria, Actinobacteriota, Bacteroidota and Ascomycota, which is consistent with previous studies showing that these phyla are the core participants in soil C and N cycling in agroecosystems (Sun et al., 2021). Proteobacteria was the main contributor to methane oxidation, nitrification and denitrification genes, and its contribution to nitrification genes was significantly higher in organic fertilizer treatments, while its contribution to denitrification genes was higher in OF0 treatment. This result indicates that fertilization can regulate the nitrogen cycling potential by shaping the abundance of specific functional groups in Proteobacteria. Actinobacteriota and Ascomycota were the main contributors to carbon degradation and nitrogen mineralization genes, and their contribution was significantly positively correlated with the organic fertilizer substitution ratio, suggesting that organic fertilizer input can select for these saprophytic microorganisms, which may be the main drivers of organic matter decomposition and nutrient mineralization in the soil (Eunjyung et al., 2023). Notably, the correlation between functional gene abundance and licorice yield and quality was not linear. OF25 and OF50 treatments had the highest licorice yield, and these two treatments also showed an optimized functional gene profile: up-regulation of carbon fixation and nitrogen mineralization genes, and down-regulation of carbon excessive degradation and nitrogen loss genetic potential genes. This result suggests that rational organic–inorganic fertilization can optimize the genetic potential of soil C and N cycling coupling at the functional gene level, which may be a potential mechanism associated with the improvement of licorice yield and quality. However, it should be emphasized that this study only established a correlation between functional gene abundance and licorice performance, and the causal relationship between microbial functional genes and the accumulation of bioactive components in licorice needs to be verified by further experiments such as microbial isolation and inoculation.

In addition, we found that soil microbial communities had high functional redundancy in C and N cycling processes: multiple microbial phyla contributed to the same functional process, and the changes in functional gene abundance were not completely consistent with the changes in the abundance of dominant taxa. This functional redundancy may be an important mechanism for the stability of soil nutrient cycling processes under environmental disturbances (Ushio et al., 2013). For example, although the abundance of Actinobacteriota decreased under OF0 treatment, the abundance of carbon degradation genes did not decrease significantly, which may be due to the functional compensation of other taxa such as Firmicutes. This result indicates that community composition can constrain or decouple the relationship between gene abundance and actual ecosystem function, and the functional redundancy of microbial communities should be fully considered when evaluating the effects of fertilization on soil nutrient cycling.

### 4.3 Niche differentiation of functional genes between bulk and rhizosphere soils

Our results showed that soil compartment (bulk vs. rhizosphere) was the primary factor driving the variation of microbial functional gene profiles, with a much stronger influence than fertilization regime (explaining 62.87% vs. 11.10% of the total variance). This result is consistent with numerous previous studies showing that rhizosphere effects can strongly shape the functional composition of soil microbial communities, and the influence of plant–microbe interactions on microbial functions is often greater than that of agricultural management measures (Ashish et al., 2024).

The rhizosphere is a hot spot of microbial activity, and root exudates can provide abundant labile carbon substrates for rhizosphere microorganisms, leading to significant differences in functional gene profiles between rhizosphere and bulk soils. We found that the response of functional genes to fertilization showed significant niche differentiation: organic fertilizer substitution mainly affected the carbon cycling functional profile in bulk soil, while it mainly affected the nitrogen cycling functional profile in rhizosphere soil. This may be because organic fertilizer input directly alters the substrate composition in bulk soil, thus shaping the carbon degradation functional profile of bulk microbial communities; while in the rhizosphere, the nitrogen demand of licorice roots drives the response of nitrogen cycling functional genes to fertilization (Tong et al., 2025). In addition, nitrogen mineralization-related genes were more abundant in the rhizosphere under high organic substitution ratio treatments, which may be because organic fertilizer promotes root exudation, and selects for rhizosphere microorganisms with strong nitrogen mineralization capacity, thus improving the nitrogen supply efficiency in the rhizosphere microhabitat (Zhang et al., 2022).

### 4.4 Limitations and future research directions

Based on our research findings, we comprehensively revealed the interaction model of licorice-soil-microorganisms under rational fertilization, as well as its influencing mechanism on carbon and nitrogen cycling (Figure 5). We also proposed optimized fertilization strategies for the sustainable cultivation of licorice. The 50% organic–inorganic combined fertilization (OF50) is recommended as the optimal regime, as it balanced soil C–N cycling genetic potential by inhibiting excessive carbon degradation and nitrogen loss genes while enhancing carbon fixation and nitrogen mineralization. Given the dominant effect of soil compartment, targeted organic fertilizer application in the rhizosphere zone is suggested to improve nutrient use efficiency. Long-term moderate organic–inorganic fertilization is also advised to maintain the stability of soil C–N cycling and microbial functional redundancy, which benefits soil health and sustainable licorice production in arid planting areas. However, several limitations of this study should be noted. First, this study was based on samples collected at a single harvest stage, and the effects of fertilization on microbial functional genes may show significant temporal dynamics during the licorice growth period. Multi-year and multi-stage monitoring is needed to confirm the stability of the observed patterns. Second, this study used metagenomic sequencing to characterize the abundance of functional genes, which only reflects the genetic potential of microbial nutrient cycling, but cannot directly reflect gene expression, enzyme activity, and the actual rate of biogeochemical processes. In the absence of transcriptomic, proteomic, enzymatic activity, and in-situ process rate measurements, the causal relationship between gene abundance changes and actual nutrient cycling processes cannot be determined. Third, the biological significance of some functional genes was interpreted in combination with existing studies, but the actual function of these genes in the licorice cultivation soil needs to be verified by further experimental evidence. For example, *GDH* is mainly involved in the intracellular nitrogen metabolism of microorganisms, and its role in soil nitrogen mineralization process is affected by multiple environmental factors such as pH, oxygen availability, and C/N ratio, so it cannot be directly regarded as the driver of soil nitrogen mineralization rate.

Future research should focus on the following directions: (1) Combine metagenomics, metatranscriptomics, and enzymatic activity measurements to link the genetic potential, expression activity, and actual process rate of microbial nutrient cycling; (2) Conduct in-situ monitoring of greenhouse gas (CH₄, N₂O) emission fluxes to verify the actual environmental effects of fertilization-induced changes in functional gene abundance; (3) Carry out long-term location experiments to explore the long-term effects of rational fertilization on soil microbial functional stability and the sustainability of licorice cultivation system.

## 5 Conclusion

In summary, this study reveals how rational fertilization influences soil microbial functional genes to balance crop productivity and environmental sustainability. Key findings include:

1. Rational organic–inorganic combined fertilization (OF50 in particular) optimizes the genetic potential of soil C–N coupling cycling, by upregulating the abundance of genes related to carbon degradation (*pel*, *cbh*), carbon fixation (*pccA*) and *GDH*-associated nitrogen mineralization genetic potential, and downregulating methanogenesis (*mttA*) and denitrification (*nirK*) genes, thus enhancing the genetic potential of soil nutrient supply and reducing the genetic potential of nutrient loss for high-quality licorice cultivation.
2. Soil compartment (bulk vs. rhizosphere) is the dominant driver of microbial functional gene profile variation in the short term, with a stronger effect than fertilization; C and N cycling genes show distinct niche differentiation in their fertilization responses between the two soil habitats, a key consideration for medicinal plant fertilization management.
3. C and N cycling functional genes in licorice soils are mainly derived from Proteobacteria, Actinobacteriota, Bacteroidota and Ascomycota. Fertilization modulates soil nutrient cycling potential by shaping these core functional taxa, and high microbial functional redundancy contributes to the stability of soil nutrient cycling processes.

## Data Availability

The datasets presented in this study can be found in online repositories. The names of the repository/repositories and accession number(s) can be found in the article/Supplementary material.
