# Supplementary material for: Enhancement of genetic potential for soil carbon and nitrogen cycling by organic fertilizer substitution improves the ecological environment for licorice cultivation
Source: Front Microbiol. 2026 May 12;17:1758116. doi: 10.3389/fmicb.2026.1758116 (PMC13201424; doi:10.3389/fmicb.2026.1758116)
Supplement: Supplementary file 1 [file Table_1.docx]

**Supplementary Material**

**
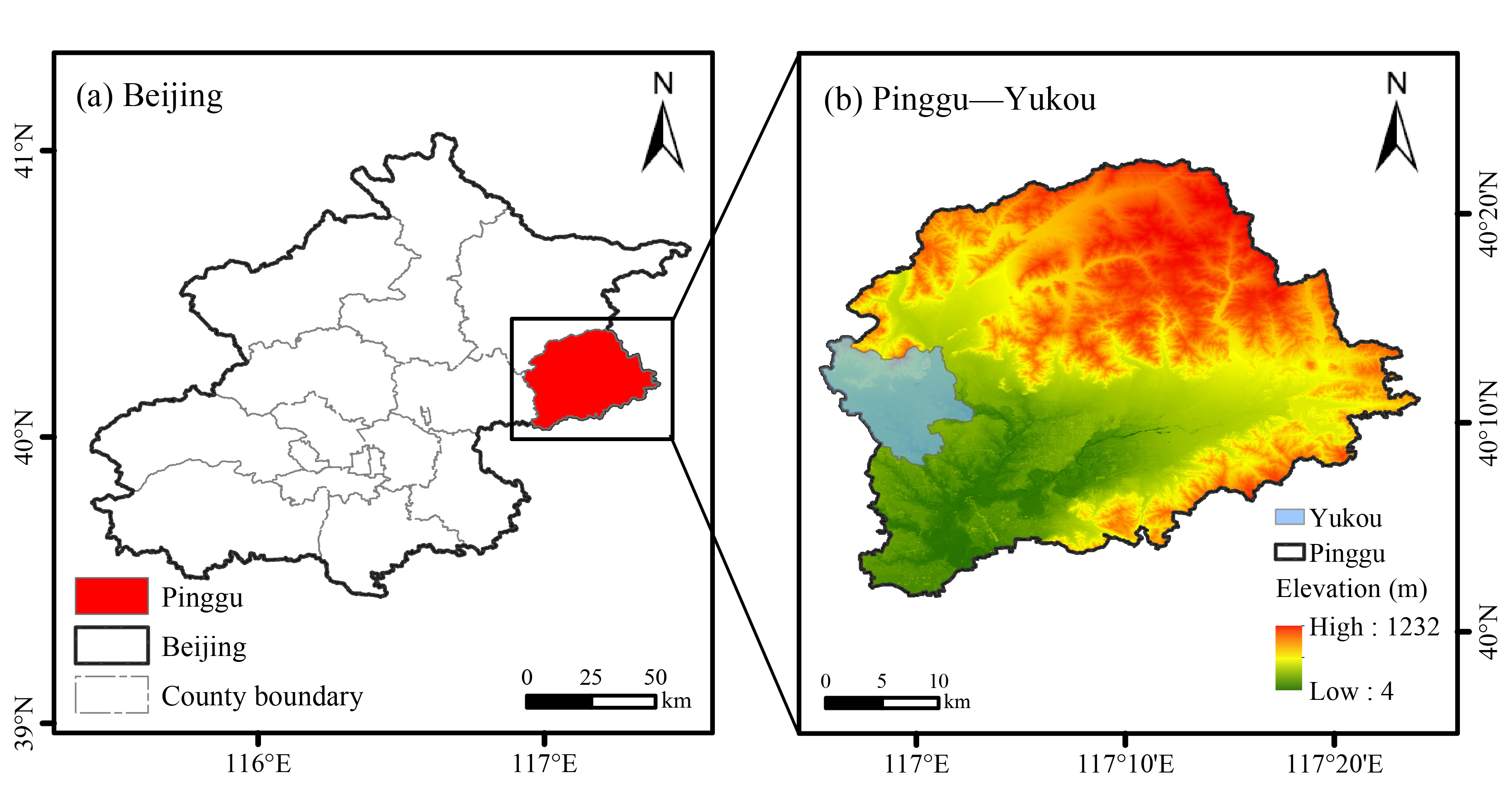
**

**Fig. S1 Location of the experimental site in Pinggu District, Beijing, China.** (a) Beijing administrative map with Pinggu District highlighted in red. (b) Topographic map of Pinggu District, marking the study site in Yukou Town (light blue area)

**Table S1**

Summary of metagenomic sequencing data and microbial community diversity indices

| Sample | Total_Reads | Q20% | Q30% | Total Input Reads | host | Contigs | ORFs | Genus-Shannon | Genus-Simpson | kegg-Shannon | kegg-Simpson | Species-Shannon | Species-Simpson |
| --- | --- | --- | --- | --- | --- | --- | --- | --- | --- | --- | --- | --- | --- |
| OF0-1-R | 64392840 | 98.62 | 95.81 | 32196420 | 3647180 | 268659 | 274296 | 8.5798 | 0.9885 | 10.2206 | 0.9984 | 10.8598 | 0.9968 |
| OF0-2-R | 66006432 | 98.14 | 94.6 | 33003216 | 2946093 | 200612 | 254099 | 8.6955 | 0.9892 | 10.3193 | 0.9986 | 10.9913 | 0.9968 |
| OF0-3-R | 66637904 | 98.94 | 96.61 | 33318952 | 4247948 | 172939 | 269605 | 8.4924 | 0.9861 | 10.2712 | 0.9985 | 10.9387 | 0.9968 |
| OF25-1-R | 68971896 | 98.74 | 96.18 | 34485948 | 4044722 | 190505 | 236698 | 8.6087 | 0.9869 | 10.3283 | 0.9986 | 10.9789 | 0.9972 |
| OF25-2-R | 71728178 | 98.72 | 96.06 | 35864089 | 3155967 | 170051 | 344350 | 8.5887 | 0.986 | 10.3548 | 0.9986 | 11.0451 | 0.9973 |
| OF25-3-R | 65814666 | 98.31 | 94.81 | 32907333 | 2053290 | 187529 | 358796 | 8.6024 | 0.9869 | 10.3729 | 0.9986 | 11.1096 | 0.9975 |
| OF50-1-R | 75048468 | 98.94 | 96.6 | 37524234 | 4669401 | 160545 | 317810 | 8.5513 | 0.985 | 10.3056 | 0.9985 | 10.9423 | 0.9965 |
| OF50-2-R | 74763716 | 99.17 | 97.33 | 37381858 | 5550617 | 231212 | 308505 | 8.3971 | 0.981 | 10.2763 | 0.9985 | 10.7046 | 0.995 |
| OF50-3-R | 67504128 | 98.7 | 95.97 | 33752064 | 2131460 | 236709 | 288360 | 8.43 | 0.9818 | 10.3656 | 0.9986 | 10.8074 | 0.9962 |
| OF75-1-R | 78075196 | 98.69 | 96.01 | 39037598 | 3502706 | 220412 | 317247 | 8.633 | 0.9882 | 10.2682 | 0.9985 | 10.9796 | 0.9972 |
| OF75-2-R | 81906896 | 99.11 | 97.14 | 40953448 | 5594899 | 216752 | 422165 | 8.5976 | 0.9851 | 10.2784 | 0.9985 | 10.8221 | 0.9956 |
| OF75-3-R | 78955542 | 97.33 | 93.93 | 39477771 | 4527092 | 192888 | 312338 | 8.5694 | 0.9832 | 10.2888 | 0.9985 | 10.7745 | 0.995 |
| OF100-1-R | 76506372 | 99.13 | 97.2 | 38253186 | 6267447 | 221136 | 371541 | 8.3593 | 0.9845 | 10.3578 | 0.9986 | 10.9603 | 0.997 |
| OF100-2-R | 84678268 | 99.13 | 97.21 | 42339134 | 5775010 | 289387 | 399621 | 8.4551 | 0.9867 | 10.3177 | 0.9986 | 10.9798 | 0.9973 |
| OF100-3-R | 70733666 | 99.32 | 97.85 | 35366833 | 9090046 | 210790 | 206273 | 8.4666 | 0.9865 | 10.3382 | 0.9986 | 10.9778 | 0.9967 |
| CK-1-R | 81304110 | 99.06 | 96.99 | 40652055 | 6201003 | 250367 | 382466 | 8.5909 | 0.9864 | 10.2256 | 0.9985 | 10.818 | 0.996 |
| CK-2-R | 74689120 | 99.07 | 97.03 | 37344560 | 5737833 | 272758 | 291378 | 8.4757 | 0.9854 | 10.2962 | 0.9985 | 10.9018 | 0.9966 |
| CK-3-R | 71232790 | 99.28 | 97.71 | 35616395 | 8092997 | 135505 | 266228 | 8.3897 | 0.9824 | 10.3609 | 0.9986 | 10.7981 | 0.9962 |
| OF0-1-B | 70527114 | 98.35 | 95.2 | 35263557 | 2560591 | 254402 | 313642 | 8.5268 | 0.9875 | 10.1591 | 0.9984 | 10.6307 | 0.9965 |
| OF0-2-B | 91549452 | 99.09 | 97.1 | 45774726 | 6766490 | 323167 | 477834 | 8.1348 | 0.9807 | 10.0697 | 0.9983 | 10.2479 | 0.9944 |
| OF0-3-B | 84394610 | 99.06 | 96.99 | 42197305 | 5227387 | 204505 | 478254 | 8.2837 | 0.9838 | 10.0866 | 0.9983 | 10.2719 | 0.995 |
| OF25-1-B | 79277296 | 99.15 | 97.28 | 39638648 | 5537234 | 220615 | 443884 | 8.1755 | 0.9801 | 10.1089 | 0.9983 | 10.1867 | 0.9947 |
| OF25-2-B | 67731136 | 98.76 | 96.17 | 33865568 | 2908697 | 320835 | 284412 | 8.421 | 0.9817 | 10.1584 | 0.9984 | 10.4718 | 0.9949 |
| OF25-3-B | 72473234 | 99.02 | 96.85 | 36236617 | 3917305 | 331115 | 376336 | 8.42 | 0.9823 | 10.1193 | 0.9983 | 10.4281 | 0.9949 |
| OF50-1-B | 67627752 | 98.75 | 96.14 | 33813876 | 2988618 | 308554 | 422905 | 8.8005 | 0.9887 | 10.1844 | 0.9984 | 10.8058 | 0.9969 |
| OF50-2-B | 67488148 | 98.64 | 95.8 | 33744074 | 2879725 | 202084 | 305931 | 8.3577 | 0.981 | 10.1141 | 0.9983 | 10.3844 | 0.9946 |
| OF50-3-B | 64453796 | 98.33 | 95.2 | 32226898 | 2842987 | 264147 | 306281 | 8.1994 | 0.975 | 10.1379 | 0.9984 | 10.2471 | 0.993 |
| OF75-1-B | 69029268 | 98.79 | 96.23 | 34514634 | 2620177 | 287910 | 421647 | 8.4378 | 0.9853 | 10.1552 | 0.9983 | 10.5438 | 0.9962 |
| OF75-2-B | 76628616 | 99.13 | 97.25 | 38314308 | 5208083 | 218935 | 361093 | 8.1461 | 0.9777 | 10.0749 | 0.9983 | 10.1528 | 0.9938 |
| OF75-3-B | 65974882 | 98.63 | 95.77 | 32987441 | 2606752 | 218168 | 375255 | 8.1093 | 0.972 | 10.1135 | 0.9983 | 10.0681 | 0.9922 |
| OF100-1-B | 77947962 | 99.01 | 96.85 | 38973981 | 5486613 | 294210 | 416326 | 7.9722 | 0.9712 | 10.0864 | 0.9983 | 9.9541 | 0.991 |
| OF100-2-B | 62686446 | 98.71 | 96.05 | 31343223 | 2427165 | 260692 | 302855 | 8.3261 | 0.9838 | 10.1248 | 0.9984 | 10.3386 | 0.9953 |
| OF100-3-B | 75144160 | 99.02 | 96.94 | 37572080 | 6034817 | 267365 | 361313 | 8.1144 | 0.9779 | 10.1 | 0.9983 | 10.1177 | 0.9933 |
| CK-1-B | 67756276 | 99.13 | 97.21 | 33878138 | 4391743 | 291628 | 357027 | 8.1435 | 0.9807 | 10.0864 | 0.9982 | 10.166 | 0.9946 |
| CK-2-B | 77321054 | 99.13 | 97.23 | 38660527 | 4713631 | 216474 | 471438 | 7.8254 | 0.9662 | 10.0489 | 0.9982 | 10.1237 | 0.9912 |
| CK-3-B | 64480980 | 98.86 | 96.49 | 32240490 | 3199746 | 251808 | 282594 | 8.165 | 0.9782 | 10.103 | 0.9983 | 10.1819 | 0.9938 |

**Table S2**

PERMANOVA of sampling location and fertilization regime under KEGG genes

| **Group dispersion** | **Df** | **Sums of squares** | **Mean squares** | **F.Model** | **Variation (R^2^)** | **Pr (>F)** |
| --- | --- | --- | --- | --- | --- | --- |
| Location | 1 | 0.119773617 | 0.119773617 | 57.87873793 | 0.628689882 | **0.001** |
| Fertilization | 1 | 0.020924390 | 0.020924390 | 10.11138602 | 0.110983180 | **0.033** |
| Location-Fertilization | 1 | 0.002426534 | 0.002426534 | 1.172584789 | 0.012736839 | 0.309 |
| Residuals | 32 | 0.066220444 | 0.002069389 |  | 0.247590099 |  |
| Total | 35 | 0.190513034 |  |  | 1 |  |

**Table S3**

KEGG genes screened for functional genes related to the C and N cycle

| **KEGG orthology number** | **Gene name** | **Encoded protein [EC]** |
| --- | --- | --- |
| **Carbon degradation** | | |
| **Starch** | | |
| K00705 | malQ | 4-alpha-glucanotransferase [EC:2.4.1.25] |
| K01187 | malZ | alpha-glucosidase [EC:3.2.1.20] |
| K01214 | ISA | isoamylase [EC:3.2.1.68] |
| **Pectin** | | |
| K01728 | pel | pectate lyase [EC:4.2.2.2] |
| K06965 | PELO | protein pelota |
| K19551 | plC | pectate lyase C [EC:4.2.2.2 4.2.2.10] |
| K21006 | pelA | polysaccharide biosynthesis protein PelA |
| K21007 | pelB | polysaccharide biosynthesis protein PelB |
| K21008 | pelC | polysaccharide biosynthesis protein PelC |
| K21009 | pelD | polysaccharide biosynthesis protein PelD |
| K21011 | pelF | polysaccharide biosynthesis protein PelF |
| K21012 | pelG | polysaccharide biosynthesis protein PelG |
| K22539 | PLY | pectate lyase [EC:4.2.2.2] |
| K01057 | PGLS | 6-phosphogluconolactonase [EC:3.1.1.31] |
| K07404 | pgl | 6-phosphogluconolactonase [EC:3.1.1.31] |
| **Cellulose** | | |
| K19668 | CBH2 | cellulose 1,4-beta-cellobiosidase [EC:3.2.1.91] |
| K01225 | CBH1 | cellulose 1,4-beta-cellobiosidase [EC:3.2.1.91] |
| K01442 | cbh | choloylglycine hydrolase [EC:3.5.1.24] |
| K01222 | celF | 6-phospho-beta-glucosidase [EC:3.2.1.86] |
| **Chitin** | | |
| K17244 | chiE | putative chitobiose transport system su*B. subtilis*trate |
| K17245 | chiF | putative chitobiose transport system permease protein |
| K17246 | chiG | putative chitobiose transport system permease protein |
| K01183 | Chi | chitinase [EC:3.2.1.14] |
| K00698 | CHS1 | chitin synthase [EC:2.4.1.16] |
| **Hemicellulose** | | |
| K01709 | rfbG | CDP-glucose 4,6-dehydratase [EC:4.2.1.45] |
| K01710 | rfbB | dTDP-glucose 4,6-dehydratase [EC:4.2.1.46] |
| K01805 | xylA | xylose isomerase [EC:5.3.1.5] |
| K10216 | dmpD | 2-hydroxymuconate-semialdehyde hydrolase [EC:3.7.1.9] |
| K10543 | xylF | D-xylose transport system su*B. subtilis*trate-binding protein |
| K10544 | xylH | D-xylose transport system permease protein |
| K01821 | praC | 4-oxalocrotonate tautomerase [EC:5.3.2.6] |
| **Carbon fixation** | | |
| **Multiple systems** | | |
| K01962 | accA | acetyl-CoA carboxylase carboxyl transferase subunit alpha [EC:6.4.1.2 2.1.3.15] |
| K01895 | ACSS1 | acetyl-CoA synthetase [EC:6.2.1.1] |
| K01965 | PCCA | propionyl-CoA carboxylase alpha chain [EC:6.4.1.3] |
| K01966 | PCCB | propionyl-CoA carboxylase beta chain [EC:6.4.1.3 2.1.3.15] |
| K11263 | bccA | acetyl-CoA/propionyl-CoA carboxylase, biotin carboxylase, biotin carboxyl carrier protein [EC:6.4.1.2 6.4.1.3 6.3.4.14] |
| K00030 | IDH3 | isocitrate dehydrogenase (NAD+) [EC:1.1.1.41] |
| K00031 | IDH1 | isocitrate dehydrogenase [EC:1.1.1.42] |
| K05824 | LYS12 | homoisocitrate dehydrogenase [EC:1.1.1.87] |
| K00174 | korA | 2-oxoglutarate/2-oxoacid ferredoxin oxidoreductase subunit alpha [EC:1.2.7.3 1.2.7.11] |
| K00175 | korB | 2-oxoglutarate/2-oxoacid ferredoxin oxidoreductase subunit beta [EC:1.2.7.3 1.2.7.11] |
| K00176 | korD | 2-oxoglutarate ferredoxin oxidoreductase subunit delta [EC:1.2.7.3] |
| K00177 | korC | 2-oxoglutarate ferredoxin oxidoreductase subunit gamma [EC:1.2.7.3] |
| **Reductive acetyl-CoA pathway** | | |
| K01938 | fhs | formate--tetrahydrofolate ligase [EC:6.3.4.3] |
| **Calvin cycle** | | |
| K01601 | rbcL | ribulose-bisphosphate carboxylase large chain [EC:4.1.1.39] |
| K01602 | rbcS | ribulose-bisphosphate carboxylase small chain [EC:4.1.1.39] |
| **Calvin cycle** | | |
| K01601 | cbbL | ribulose bisphosphate carboxylase large chain [EC:4.1.1.39] |
| K01602 | cbbR | ruBisCO operon transcriptional regulator |
| **Methane metabolism** | | |
| **Methane oxidation** | | |
| K10944 | pmoA-amoA | methane/ammonia monooxygenase subunit A [EC:1.14.18.3 1.14.99.39] |
| K10945 | pmoB-amoB | methane/ammonia monooxygenase subunit B |
| K10946 | pmoC-amoC | methane/ammonia monooxygenase subunit C |
| **Methanogenesis** | | |
| K00577 | mttA | tetrahydromethanopterin S-methyltransferase subunit A [EC:2.1.1.86] |
| K07670 | mtrA | two-component system, OmpR family, response regulator MtrA |
| K14083 | mttB | trimethylamine---corrinoid protein Co-methyltransferase [EC:2.1.1.250] |
| K07654 | mtrB | two-component system, OmpR family, sensor histidine kinase MtrB [EC:2.7.13.3] |
| **Nitrogen fixation** | | |
| K02584 | nifA | Nif-specific regulatory protein |
| K02586 | nifD | nitrogenase molybdenum-iron protein alpha chain [EC:1.18.6.1] |
| K02594 | nifV | homocitrate synthase NifV [EC:2.3.3.14] |
| K02588 | nifK | nitrogenase molybdenum-iron protein beta chain [EC:1.18.6.1] |
| K02591 | nifV | homocitrate synthase NifV [EC:2.3.3.14] |
| **Denitrification** | | |
| K00370 | narG | nitrate reductase / nitrite oxidoreductase, alpha subunit [EC:1.7.5.1 1.7.99] |
| K00371 | narH | nitrate reductase / nitrite oxidoreductase, beta subunit [EC:1.7.5.1 1.7.99] |
| K00368 | nirK | nitrite reductase (NO-forming) [EC:1.7.2.1] |
| K15864 | nirS | nitrite reductase (NO-forming) [EC:1.7.2.1 1.7.99.1] |
| K04561 | norB | nitric oxide reductase subunit B [EC:1.7.2.5] |
| K02305 | norC | nitric oxide reductase subunit C |
| K00376 | nosZ | nitrous-oxide reductase [EC:1.7.2.4] |
| **Nitrification** | | |
| K10944 | pmoA-amoA | methane monooxygenase subunit A [EC:1.14.18.3 1.14.99.39] |
| K10945 | pmoB-amoB | methane/ammonia monooxygenase subunit B |
| K10946 | pmoC-amoC | methane/ammonia monooxygenase subunit C |
| **Assimilatory nitrate reduction (ANRA)** | | |
| K00372 | nasC, nasA | assimilatory nitrate reductase catalytic subunit [EC:1.7.99.-] |
| K02575 | NRT | MFS transporter, NNP family, nitrate/nitrite transporter |
| K15578 | nrtC | nitrate/nitrite transport system ATP-binding protein [EC:7.3.2.4] |
| K00366 | nirA | ferredoxin-nitrite reductase [EC:1.7.7.1] |
| **Dissimilatory nitrate reduction (DNRA)** | | |
| K02567 | napA | nitrate reductase (cytochrome) [EC:1.9.6.1] |
| K02568 | napB | nitrate reductase (cytochrome), electron transfer subunit |
| K03385 | nrfA | nitrite reductase (cytochrome c-552) [EC:1.7.2.2] |
| **Anammox/Hydroxylamine oxidation** | | |
| K10535 | hao | hydroxylamine dehydrogenase [EC:1.7.2.6] |
| **Nitrogen degradation** | | |
| K01428 | ureC | urease subunit alpha [EC:3.5.1.5] |
| K15371 | GDH2 | glutamate dehydrogenase [EC:1.4.1.2] |
| K19813 | GDH1 | glucose dehydrogenase [EC:1.1.5.9] |
| K22969 | GDH3 | glucose 1-dehydrogenase [EC:1.1.1.47] |
| K00012 | UGDH | UDPglucose 6-dehydrogenase [EC:1.1.1.22] |
| K00058 | serA | D-3-phosphoglycerate dehydrogenase / 2-oxoglutarate reductase [EC:1.1.1.95 1.1.1.399] |
| K00109 | L2HGDH | 2-hydroxyglutarate dehydrogenase [EC:1.1.99.2] |
| K00261 | GLUD1_2 | glutamate dehydrogenase (NAD(P)+) [EC:1.4.1.3] |
| K00262 | GDHA | glutamate dehydrogenase (NADP+) [EC:1.4.1.4] |
| K00315 | DMGDH | dimethylglycine dehydrogenase [EC:1.5.8.4] |
